# Successful Treatment of Atlantoaxial Subluxation in an Adolescent Patient with BrachytelephalangicChondrodysplasia Punctata

**DOI:** 10.1155/2019/5974281

**Published:** 2019-01-27

**Authors:** Yoh Fujimoto, Yuki Taniguchi, Yasushi Oshima, Yoshitaka Matsubayashi, Keita Okada, Nobuhiko Haga, Sakae Tanaka

**Affiliations:** ^1^Department of Orthopedic Surgery, The University of Tokyo Hospital, Tokyo, Japan; ^2^Department of Rehabilitation Medicine, The University of Tokyo Hospital, Tokyo, Japan

## Abstract

Brachytelephalangic chondrodysplasia punctata (CDPX1) is characterized by brachytelephalangy and nasomaxillary hypoplasia, in addition to stippled epiphyses. Some reports have described infants with CDPX1 who exhibited cervical spinal stenosis. However, the natural course of cervical spinal lesions in this condition has not been elucidated. Here, we report a very rare adolescent case of CDPX1, which demonstrated progressive myelopathy caused by atlantoaxial subluxation and a subsequent retroodontoid pseudotumor, successfully treated with surgery. Our case highlights a new clinically important fact that upper cervical spinal lesions in CDPX1 can deteriorate even after childhood and thus need close monitoring.

## 1. Introduction

Chondrodysplasia punctata (CDP) is a group of dysplasias characterized by stippled calcifications within the epiphyses in infancy [1]. Several types of CDP have been reported, which include rhizomelic CDP types 1-3, Conradi-Hünermann type, tibial-metacarpal type, CHILD (congenital hemidysplasia with ichthyosiform erythroderma and limb defects) syndrome, and brachytelephalangic CDP [2]. Brachytelephalangic CDP, also known as X-linked CDP 1 (CDPX1, OMIM 302950), is characterized by stippled epiphyses, brachytelephalangy (shortening of the distal phalanges), and nasomaxillary hypoplasia and was first described by Maroteaux in 1989 [3]. Regarding spinal lesions in CDPX1, cervical spinal stenosis in infants has been reported; however, no report has described cervical spinal lesions in adolescents [4–8]. In this case report, we describe a very rare case of an adolescent patient who demonstrated progressive myelopathy caused by atlantoaxial subluxation associated with CDPX1 and was successfully treated with surgery.

## 2. Case Presentation

### 2.1. Presentation and Examination

**Figure 1:**
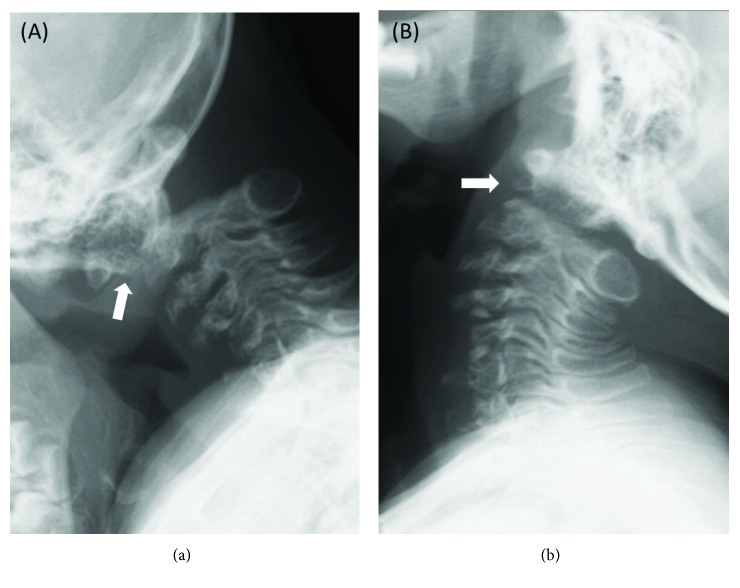
(a, b) Lateral plain radiographs of the cervical spine in flexion and extension at the age of 5 years. Multiple dysplastic vertebral bodies and atlantoaxial instability with os odontoideum are evident (*white arrows*).

**Figure 2:**
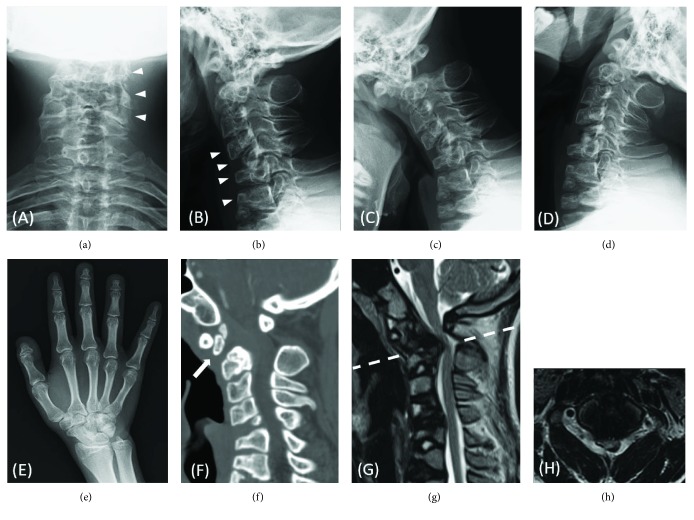
(a, b) Preoperative anterior-posterior and lateral plain radiographs of the cervical spine revealing multiple dysplastic vertebral bodies (*white arrowheads*). (c, d) Preoperative lateral plain radiographs of the cervical spine clearly demonstrating unstable atlantoaxial subluxation. (e) An anterior-posterior plain radiograph of the right hand revealing typical shortening of the distal phalanges (brachytelephalangy) of the thumb and middle and ring fingers. (f) Preoperative sagittal reconstructed computed tomography showing the segmented os odontoideum (*white arrow*). (g) Preoperative sagittal T2-weighted magnetic resonance (MR) images showing a severely compressed spinal cord at the C1/2 level with signs of myelomalacia inside the cord. A low-intensity retroodontoid mass is evident on the T2-weighted MR images. (h) Preoperative axial T2-weighted MR image at the C1/2 level revealing a severely compressed spinal cord. The white dotted lines in (g) indicate the level of (h).

The patient first presented to our department at the age of 6 years for the treatment of atlantoaxial subluxation associated with CDPX1 diagnosed previously. Physical examination revealed nasal hypoplasia, hearing loss, and brachytelephalangy, which are characteristic of CDPX1. His family history was nonremarkable. Although the stippled calcifications in the epiphysis had already disappeared, cervical radiography showed highly dysplastic vertebrae (Figure 1). He also had type 8 glycogen storage disease; however, it had resolved at the age of 3 years. At his first visit to our department, he showed no signs of myelopathy, and magnetic resonance imaging (MRI) showed no cervical spinal canal stenosis, although atlantoaxial instability due to os odontoideum was already apparent on radiography (Figure 1). Subsequently, we started follow-up at the outpatient clinic every year. At the age of 16 years, the patient complained of progressive gait disturbance and mild upper extremity weakness due to cervical myelopathy. Cervical radiography revealed multiple dysplastic vertebral bodies (Figures 2(a) and 2(b)). Dynamic radiography revealed unstable atlantoaxial subluxation (Figures 2(c) and 2(d)). A plain radiograph of the right hand showed typical shortening of the distal phalanges (brachytelephalangy) of the thumb and middle and ring fingers (Figure 2(e)). Sagittal reconstruction computed tomography showed segmented os odontoideum (Figure 2(f)). MRI demonstrated severe spinal cord compression at the C1/2 level with a sign of myelomalacia inside the cord (Figures 2(g) and 2(h)). MRI also revealed a retroodontoid mass, which was thought to be a pseudotumor caused by upper cervical instability (Figures 2(g) and 2(h)) [9].

### 2.2. Surgery

Because the myelopathy was progressive, we performed surgery. Although the spinal cord compression was caused by atlantoaxial instability, posterior O-C2 fusion and resection of the C1 posterior arch were performed because the C1 lateral mass was too small for screw insertion. After the surgery, the neurological deficit gradually recovered, and at 1 month after surgery, his gait disturbance improved.

**Figure 3:**
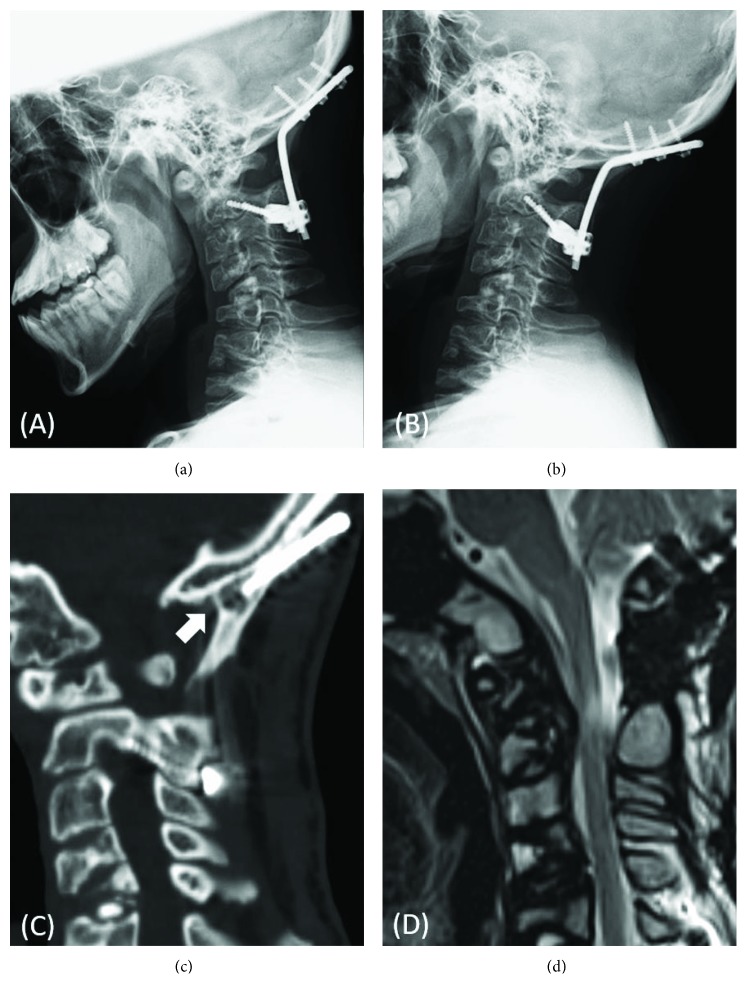
(a, b) Lateral radiographs of the cervical spine in flexion and extension at 1 year after surgery demonstrating a stabilized O-C2 segment. (c) Sagittal reconstruction computed tomography taken at 1 year after surgery showing solid fusion between the grafted iliac bone and occipital bone (*white arrow*). (d) Sagittal T2-weighted magnetic resonance image taken at 1 year after surgery revealing a well-decompressed spinal cord with marked regression of the retroodontoid pseudotumor.

At 1 year after surgery, he showed no myelopathy. Dynamic radiography revealed a stabilized O-C2 segment, although slight loosening of the pedicle screws at C2 was apparent (Figures 3(a) and 3(b)). Sagittal reconstruction computed tomography showed solid fusion between the grafted iliac bone and occipital bone (Figure 3(c)). MRI revealed a well-decompressed spinal cord with marked regression of the retroodontoid mass, which indicated that this mass was possibly a pseudotumor (Figure 3(d)).

## 3. Discussion

Maroteaux was the first to report on CDPX1 in detail [3]. The clinical presentation of CDPX1 is characterized by a short stature and a characteristic dysmorphic face with a depressed nasal bridge and hypoplasia of the distal phalanges (brachytelephalangy) [3]. As stippled epiphyses usually disappear in the first two years of life in cases with CDP, Maroteaux emphasized the importance of the phalangeal anomaly in the diagnosis of CDPX1 at an age at which epiphyseal stippling is no longer present [3]. Because our patient was first referred to our department at the age of 6 years, typical stippled epiphyses had already disappeared on the radiographs; hence, radiography of the hand was helpful for the confirmation of the diagnosis of CDPX1, which had already been made by his previous doctors on the basis of typical radiographic findings, including stippled epiphyses in infancy.

Maroteaux reported that CDPX1 had a good prognosis, but a few reports have described lethal cases of CDPX1 in infants, which were complicated by severe cervical myelopathy due to upper cervical stenosis [4, 5]. Hence, it is crucial to adequately assess for cervical spinal lesions in the management of CDPX1. Nino et al. reported that cervical spinal abnormality is found in about 20% of CDPX1 cases; however, no report has clarified the natural history of cervical spinal lesions [7]. Considering the fact that all previous surgeries for cervical spinal lesions in CDPX1 were performed in infancy or early childhood, it is suggested that most cervical spinal lesions in CDPX1 cases can rapidly deteriorate until early childhood [5, 6, 8, 10–12]. Our case report provides clinically important insight that upper cervical spinal lesions can deteriorate even after childhood, thus necessitating close monitoring in childhood and adolescence.

Cervical spinal lesions in CDPX1 can involve both upper cervical and subaxial lesions. In their case series, Morota et al. reported that among seven patients with CDPX1 who underwent cervical spinal surgery in infancy or early childhood, six had upper cervical lesions and one had subaxial stenosis [6]. In their thorough review of the literature written in the English language on cervical spine disease in 44 cases of CDP, Vogel and Menezes reported 16 cases of CDPX1 with cervical spinal lesions, including nine cases with upper cervical stenosis and three cases with subaxial stenosis [10]. Hence, clinicians have to monitor the entire cervical spine during follow-up of CDPX1 cases.

Vogel and Menezes reported the long-term outcome of surgery for a cervical spinal lesion in a case of coumarin embryopathy in detail [10]. Because coumarin embryopathy, which results from coumarin exposure during pregnancy, is considered a phenocopy of CDP, this case suggests the natural history of cervical spinal lesions in CDPX1 owing to their similar phenotypic characteristics. In this case, O-C3 fusion with halo cervical traction was performed at the age of 7 years for progressive neurological deterioration due to atlantoaxial instability, followed by transoral resection of C2-4 vertebral bodies at the age of 9 years, and posterolateral C3-4 fusion at the age of 10 years. The patient recovered well for 22 years after the last surgery [10]. This case shows that CDPX1 patients with cervical spinal lesions can expect a good long-term neurological prognosis if their cervical spinal lesions are adequately managed.

Because CDPX1 patients have inherent structural weakness of the bone, the long-term outcome after instrumentation surgery is concerning. With regard to cervical spinal lesions in CDPX1 patients, until now there have been only two reported cases of cervical instrumentation surgery [6, 8]. In one case, surgery was performed at the age of 18 months, and in the other case, instrumentation surgery was performed at the age of 4 years. In both cases, solid fusion was confirmed at 6 and 9 months after surgery, respectively, although in the latter case, the instrumentation was removed after verification of fusion because of deep wound infection. In contrast, in our adolescent case, slight loosening of the instrumentation at 1 year after surgery was observed, although clinically insignificant, which may suggest innate poor bone quality of CDPX1 cases in the adolescent period. Further investigation of adolescent CDPX1 cases is needed to clarify this point.

The potential of bone fusion in CDPX1 is unknown. Because this disease is characterized by the failure of the endochondral ossification process, which also plays an essential role in fracture healing, the bone fusion process may be impaired in this disease. In our case, solid fusion between the grafted iliac bone and occipital bone was verified by computed tomography at 1 year after surgery, which suggests that this disease has minimal or no impact on the bone fusion process.

In conclusion, we have reported a very rare adolescent case of CDPX1, which presented with progressive myelopathy caused by atlantoaxial instability due to os odontoideum and was successfully treated with posterior surgery. Careful radiographic and neurological follow-up is mandatory for cases of CDPX1 even after childhood.
